# Depolarization of sperm membrane potential is a common feature of men with subfertility and is associated with low fertilization rate at IVF

**DOI:** 10.1093/humrep/dew056

**Published:** 2016-04-06

**Authors:** Sean G. Brown, Stephen J. Publicover, Steven A. Mansell, Polina V. Lishko, Hannah L. Williams, Mythili Ramalingam, Stuart M. Wilson, Christopher L.R. Barratt, Keith A. Sutton, Sarah Martins Da Silva

**Affiliations:** 1 School of Science, Engineering and Technology, Abertay University, DundeeDD11HG, UK; 2 School of Biosciences, The University of Birmingham, BirminghamB15 2TT, UK; 3 Reproductive and Developmental Biology, School of Medicine, Ninewells Hospital and Medical School, University of Dundee, DundeeDD19SY, UK; 4 Department of Molecular and Cell Biology, University of California, Berkeley, CA, USA; 5 Wolfson Research Institute, School of Medicine, Pharmacy and Health, University of Durham, Queen's Campus, Stockton on Tees TS17 6BH, UK

**Keywords:** patch clamp electrophysiology, potassium channel, spermatozoa, IVF, male infertility, Slo1, Slo3, sperm dysfunction, CatSper

## Abstract

**STUDY QUESTION:**

Are significant abnormalities in outward (K^+^) conductance and resting membrane potential (*V*_m_) present in the spermatozoa of patients undertaking IVF and ICSI and if so, what is their functional effect on fertilization success?

**SUMMARY ANSWER:**

Negligible outward conductance (≈5% of patients) or an enhanced inward conductance (≈4% of patients), both of which caused depolarization of *V*_m_, were associated with a low rate of fertilization following IVF.

**WHAT IS KNOWN ALREADY:**

Sperm-specific potassium channel knockout mice are infertile with defects in sperm function, suggesting that these channels are essential for fertility. These observations suggest that malfunction of K^+^ channels in human spermatozoa might contribute significantly to the occurrence of subfertility in men. However, remarkably little is known of the nature of K^+^ channels in human spermatozoa or the incidence and functional consequences of K^+^ channel defects.

**STUDY DESIGN, SIZE AND DURATION:**

Spermatozoa were obtained from healthy volunteer research donors and subfertile IVF and ICSI patients attending a hospital assisted reproductive techniques clinic between May 2013 and December 2015. In total, 40 IVF patients, 41 ICSI patients and 26 normozoospermic donors took part in the study.

**PARTICIPANTS/MATERIALS, SETTING, METHODS:**

Samples were examined using electrophysiology (whole-cell patch clamping). Where abnormal electrophysiological characteristics were identified, spermatozoa were further examined for Ca^2+^ influx induced by progesterone and penetration into viscous media if sufficient sample was available. Full exome sequencing was performed to specifically evaluate potassium calcium-activated channel subfamily M α 1 (*KCNMA1),* potassium calcium-activated channel subfamily U member 1 *(KCNU1)* and leucine-rich repeat containing 52 (*LRRC52)* genes and others associated with K^+^ signalling. In IVF patients, comparison with fertilization rates was done to assess the functional significance of the electrophysiological abnormalities.

**MAIN RESULTS AND THE ROLE OF CHANCE:**

Patch clamp electrophysiology was used to assess outward (K^+^) conductance and resting membrane potential (*V*_m_) and signalling/motility assays were used to assess functional characteristics of sperm from IVF and ICSI patient samples. The mean *V*_m_ and outward membrane conductance in sperm from IVF and ICSI patients were not significantly different from those of control (donor) sperm prepared under the same conditions, but variation between individuals was significantly greater (*P*< 0.02) with a large number of outliers (>25%). In particular, in ≈10% of patients (7/81), we observed either a negligible outward conductance (4 patients) or an enhanced inward current (3 patients), both of which caused depolarization of *V*_m_. Analysis of clinical data from the IVF patients showed significant association of depolarized *V*_m_ (≥0 mV) with low fertilization rate (*P*= 0.012). Spermatozoa with electrophysiological abnormities (conductance and *V*_m_) responded normally to progesterone with elevation of [Ca^2+^]*_i_* and penetration of viscous medium, indicating retention of cation channel of sperm (CatSper) channel function.

**LIMITATIONS, REASONS FOR CAUTION:**

For practical, technical, ethical and logistical reasons, we could not obtain sufficient additional semen samples from men with conductance abnormalities to establish the cause of the conductance defects. Full exome sequencing was only available in two men with conductance defects.

**WIDER IMPLICATIONS OF THE FINDINGS:**

These data add significantly to the understanding of the role of ion channels in human sperm function and its impact on male fertility. Impaired potassium channel conductance (Gm) and/or *V*_m_ regulation is both common and complex in human spermatozoa and importantly is associated with impaired fertilization capacity when the *V*_m_ of cells is completely depolarized.

**STUDY FUNDING/COMPETING INTEREST(S):**

The majority of the data were obtained using funding from MRC project grants (#MR/K013343/1, MR/012492/1). Additional funding was provided by NHS Tayside, TENOVUS, Chief Scientist Office NRS Fellowship and University of Abertay. The authors declare that there is no conflict of interest.

**TRIAL REGISTRATION NUMBER:**

Not applicable.

## Introduction

The membrane potential (*V*_m_) of animal cells is typically polarized (negatively charged inside), primarily by the activity of K^+^ channels. In spermatozoa, as in many cell types, *V*_m_ modulates the activity of membrane ion channels and transporters, including the sperm-specific Ca^2+^-permeable channel CatSper and voltage-gated proton channel Hv1 (Darszon *et al.*, 1999, 2011; Lishko *et al.*, 2012). Maintenance and modulation of *V*_m_ is therefore pivotal to sperm function and characterizing the expression and regulation of membrane K^+^ channels is potentially crucial to understanding the function of spermatozoa from both normal and subfertile men.

Mammalian sperm potassium channels are formed by proteins of the potassium calcium-activated channel (*KCN*) family. In mouse, sperm slowpoke homologue 3 (Slo3), a sperm-specific protein, forms the primary K^+^ channel. The pharmacology and biophysical properties of mouse sperm K^+^ channels are accurately reconstituted by co-expression of recombinant Slo3 [approved name potassium calcium-activated channel subfamily U member 1 (KCNU1)] and the auxiliary subunit LRRC52 [leucine-rich-repeat-containing protein (Navarro *et al.*, 2007; Santi *et al.*, 2010; Zeng *et al.*, 2011, 2013, 2015)]. Mice null for Slo3 or LRRC52 have markedly reduced fertility (Santi *et al.*, 2010; Zeng *et al.*, 2015) and sperm from these animals show functional impairments including depolarized values of *V*_m_, a failure to hyperpolarize during capacitation, reduced motility and lack of hyperactivation (Santi *et al.*, 2010; Zeng *et al.*, 2015).

These observations suggest that malfunction of K^+^ channels in human spermatozoa might contribute significantly to the occurrence of subfertility in men. However, patch clamp studies have shown that the complement of ion channels, their regulation and their functional significance differ significantly between human and mouse sperm (Miller *et al.*, 2015). Pharmacological studies on human sperm suggest that Slo1 is expressed and involved in setting *V*_m_ (Mannowetz *et al.*, 2013; Lopez-Gonzalez *et al.*, 2014), although some characteristics of currents measured by patch clamp resemble Slo3 (Mannowetz *et al.*, 2013; Brenker *et al.*, 2014; Mansell *et al.*, 2014). However, remarkably little is known of the nature of K^+^ channels in human spermatozoa or the incidence and functional consequences of K^+^ channel defects.

To address the question of the functional importance of K^+^ channels in human spermatozoa and the potential contribution of their malfunction to subfertility, we have used whole-cell patch clamp electrophysiology to assess the biophysical characteristics of spermatozoa from semen samples provided by 80 men attending for fertility treatment. This unique approach has allowed us to assess (i) the proportion of men undergoing treatment whose sperm show abnormalities of K^+^ channel expression/regulation of *V*_m_ (compared with sperm from normal, ‘healthy’ donors) and (ii) the association between abnormalities of K^+^ channel expression/regulation of *V*_m_ and fertilizing capacity in IVF. In this, the first study of this type, we report the prevalence, characteristics and importantly the functional consequences of abnormalities of K^+^ channel function and maintenance of *V*_m_ in human spermatozoa.

## Materials and Methods

### Experimental design

Single whole-cell sperm patch clamp recordings were made on the same day of patient treatment allowing contemporaneous assessment of K^+^ channel function in cells from the same ejaculate as that used for IVF. This was also the case for the majority of the ICSI patients; however, in some cases, patients were recruited as part of an IVF failed fertilization research clinic. Patch-clamp electrophysiology has been employed to characterize sperm membrane currents under quasi-physiological conditions (Mansell *et al.*, 2014). We employed the identical strategy in order to maximize the information obtained from each patient cell. Outward potassium currents were evoked by imposing a depolarizing voltage ramp from −92 to 68 mV. To assess the functional consequences of channel function, comparison with IVF fertilization rates was made. In specific patients of interest (those with channel conductance abnormalities) and, where sufficient semen sample was available, patient samples were also assessed for Ca^2+^ influx in response to progesterone and viscous media penetration test. Additionally, genetic screening was performed only on patient samples with conductance abnormalities who also provided informed consent.

Patients displaying significant electrophysiological abnormalities and/or examined in more detail are referred to by a single letter (A, C, D, K, X, Y).

### Experimental solutions

Standard extracellular solution: NaCl, 135 mM; KCl, 5 mM; CaCl_2_, 2 mM; MgSO_4_, 1 mM; HEPES, 20 mM; glucose, 5 mM; Na pyruvate, 1 mM; lactic acid, 10 mM; pH adjusted to 7.4 with NaOH which brought [Na^+^] to 154 mM. Standard pipette solution: NaCl, 10 mM; KCl, 18 mM; K gluconate, 92 mM; MgCl_2_, 0.5 mM, CaCl_2_, 0.6 mM; EGTA, 1 mM; HEPES, 10 mM; pH adjusted to 7.4 using KOH which brought [K^+^] to 114 mM and [Ca^2+^]*_i_* to 0.1 µM. Recordings from cells from Patient K in which effects of elevating [Ca^2+^]*_i_* were tested were obtained from a second sample using the following solutions. Pipette solution: 145 mM; KMeSO_4_, 5 mM; HEPES, 4 mM; KCl, 1 mM; BAPTA, 1 mM; EDTA, 1 mM; EGTA, 1.7 mM; CaCl_2_ pH 7.4 with KOH (final [Ca^2+^]*_i_* = 0.1 µM which is sufficient to inhibit monovalent CatSper currents (Lishko *et al*., 2011). High calcium pipette solution: 145 mM; KMeSO_4_, 5 mM; HEPES, 4 mM; KCl, 1 mM; HEDTA, 1 mM; CaCl_2_ pH 7.4 with KOH (final [Ca^2+^]*_i_* = 50 µM). Bath solution (mM): 140; KMeSo_4_, 45; HEPES, 0.1 CaCl_2_ pH 7.4 with KOH. [Ca^2+^] in buffered solutions was calculated using MaxChelator (Maxchelator.stanford.edu).

### Selection of patients and preparation of spermatozoa

Patients were selected for IVF according to clinical indications and semen quality: e.g. normal sperm concentration and motility (WHO, 2010) and ∼1 × 10^6^ progressively motile cells post-preparation (Williams *et al.*, 2015). Patients having ICSI were those with male factor infertility undergoing ICSI for the first time due to poor quality semen and/or from patients recalled following IVF treatment affected by at least one cycle total failed fertilization/very low fertilization.

Semen samples were obtained from volunteer donors with no known fertility problems with normal sperm concentration and motility. Donor samples were obtained by masturbation after 48–72 h of sexual abstinence. In accordance with established WHO criteria, all donors were shown to produce normal semen characteristics (i.e. ≥32% progressive motility; ≥40% total motility; ≥15 × 10^6^ cells/ml). Sperm cells were isolated using a 40–80% discontinuous density gradient procedure (Alasmari *et al.*, 2013a,b; Tardif *et al.*, 2014). Briefly, after 30 min of liquefaction at 37°C, up to 2 ml of semen was loaded on top of a colloidal silica suspension (Percoll, Sigma Aldrich, UK) made of 80 and 40% layers (1.5 ml each). The density gradient was then centrifuged at 300*g* for 20 min. Cells were washed in Quinn's Advantage Sperm Washing Media (SWM) (500*g*, 10 min) and resuspended in Quinn's Advantage™ Fertilization Media supplemented with human serum albumin (5%). Cells were left to capacitate at 37°C/5% CO_2_ for a minimum of 3 h prior to recording, which was a procedure similar to that used to prepare spermatozoa for IVF. For conditions similar to ICSI, once spermatozoa were washed, they were incubated in SWM at 37°C.

In the Assisted Conception Unit, commercially available media was used for sperm preparation. The spermatozoa were separated from semen by density gradient centrifugation (40:80%) using PureSperm™ (Nidacon, Molndal, Sweden) diluted with Quinns Advantage SWM, a HEPES-buffered solution (Cooper Surgical Inc., USA). After centrifugation, the pellet was washed by centrifugation at 500*g* for 10 min in 4 ml of SWM. The supernatant was discarded and the pellet resuspended in Quinns Advantage Fertilization Media (Cooper Surgical Inc.). For ICSI, once cells were washed, they were incubated in SWM at 37°C. Cells surplus to requirement were made available for research.

Patient D provided a second sample for research only 11 days after IVF treatment which was used for electrophysiological/computer-assisted sperm analysis (CASA) analysis. The mean electrophysiological data are presented from the treatment and research sample from this patient. Patients D and K had a vasectomy reversal.

### Electrophysiology

The biophysical properties of individual sperm were recorded under whole cell conditions (Lishko *et al.*, 2011; Mansell *et al.*, 2014) using borosilicate glass pipettes (10–15 MOhms) filled with standard pipette solution. Gigaohm seals were formed at the back of the head region and suction applied to achieve a whole-cell configuration. It is of note that flagellar beating was observed in all cells selected. Sperm were perfused with standard extracellular solution designed to maintain physiologically relevant ion concentrations (Mansell *et al.*, 2014). To investigate cell conductance and reversal potential, a depolarizing ramp protocol was imposed (−92 to 68 mV) over 2500 ms and membrane potential was held at −92 mV between test pulses. Analysis was done on the average response of currents evoked by successive depolarizations on an individual spermatozoon. Data were sampled at 2 kHz and filtered at 1 kHz (PClamp 10 software, Axon Instruments, USA). Post-recording analysis was conducted as described previously to adjust for liquid junction potential and normalize for cell size (Mansell *et al.*, 2014). Reversal potentials (*E*_rev_) were calculated by regression analysis of membrane current over the imposed potential range where the membrane current crosses the *x*-axis (i.e. *I* = 0). Resting membrane potential (*V*_m_) is inferred from *E*_rev_. All control data are taken from cells from at least three different donors. Membrane conductance (Gm) were derived by regression analysis of data recorded from 20 to 68 mV.

### Assessment of [Ca^2+^]_i_ signals

Approximately 4 million cells were prepared and assessed as previously described using a FLUOstar microplate reader (BMG Labtech Offenburg, Germany) (Alasmari *et al.*, 2013a). After recording for an initial control period, progesterone (3.6 μM) was added to the well. Progesterone-induced increments in the ratio of emission intensities (at 340 and 380 nm excitation) were used to quantify changes in [Ca^2+^]*_i_* concentration (Alasmari *et al.*, 2013a,b).

### Viscous media penetration test method

The penetration into viscous media test was performed as previously described (Alasmari *et al.*, 2013a,b). After a minimum of 3 h of capacitation, prepared sperm were adjusted to ∼10–20 × 10^6^/ml before addition of 3.6 µM progesterone/vehicle. Following 1 h incubation at 37°C and 5% CO_2_, cell numbers at a penetration depth of 1 cm were counted at four different focal planes using a Hamilton Thorne CASA system (Alasmari *et al.,* 2013a), and were normalized to values from parallel, untreated controls.

### Fertilization rate at IVF

Oocytes were considered normally fertilized when two pronuclei (2PN) and two distinct or fragmented polar bodies were observed. Following IVF, the fertilization rate was calculated from the number of oocytes normally fertilized divided by the total number of inseminated oocytes. The fertilization rate was calculated where four or more mature oocytes (metaphase II) were present. Low fertilization was defined as where <25% of 4 or more metaphase II oocytes were normally fertilized.

### Ethical approval

Written consent was obtained from each patient in accordance with the Human Fertilization and Embryology Authority (HFEA) Code of Practice (version 8) under local ethical approval (13/ES/0091) from the Tayside Committee of Medical Research Ethics B. Similarly, volunteer sperm donors were recruited in accordance with the HFEA Code of Practice (version 8) under the same ethical approval.

### Genetic screening

Genetic screening was only performed on patient samples with conductance abnormalities who also provided informed consent. Analysis was performed on Patients D and K. DNA extracted from blood was subjected to whole-exome sequencing as previously described (Williams *et al.*, 2015). Single-nucleotide polymorphism (SNP) and insertion or the deletion of bases (Indel) identification was performed using a standard Genome Analysis Tool Kit (GATK) workflow following bowtie alignment (McKenna *et al.*, 2010; DePristo *et al.*, 2011; Van der Auwera *et al.*, 2013). To identify mutations in loci that may influence potassium conductance, we used the Sting database (http://string-db.org/) to identify a list of candidates. Mutations with an allele frequency >0.05 (as per 1000 Genomes, http://www.1000genomes.org/) were excluded.

### Data analysis

Currents evoked by successive voltage ramps were pooled in order to obtain an average response from each spermatozoon. Statistical analysis was carried out to compare parameters between donor sperm and sperm from individual patients when possible. For plotted ramp current–voltage (*I*–*V*) relationships, the mean current per millivolt was calculated and mean ± SEM are presented. Data were analysed using Microsoft Excel™ or GraphPad Prism™ (version 5, GraphPad Software Inc.). The normality of *V*_m_ data was confirmed both by comparison of frequency distributions with normal distribution generated from sample mean and standard deviation and by generation of quantile–quantile (*Q*–*Q*) plots. Statistical significance was determined using the *F*-test (Variance ratio), Student’s unpaired t-test or analysis of variance/Dunnets *post hoc* test as appropriate. Data are presented as mean ± SEM with *P*< 0.05 indicative of statistical significance. *P< 0.05, **P< 0.01, ***P< 0.001.

## Results

### Voltage ramp-induced currents in donors and patients

**Figure 1:**
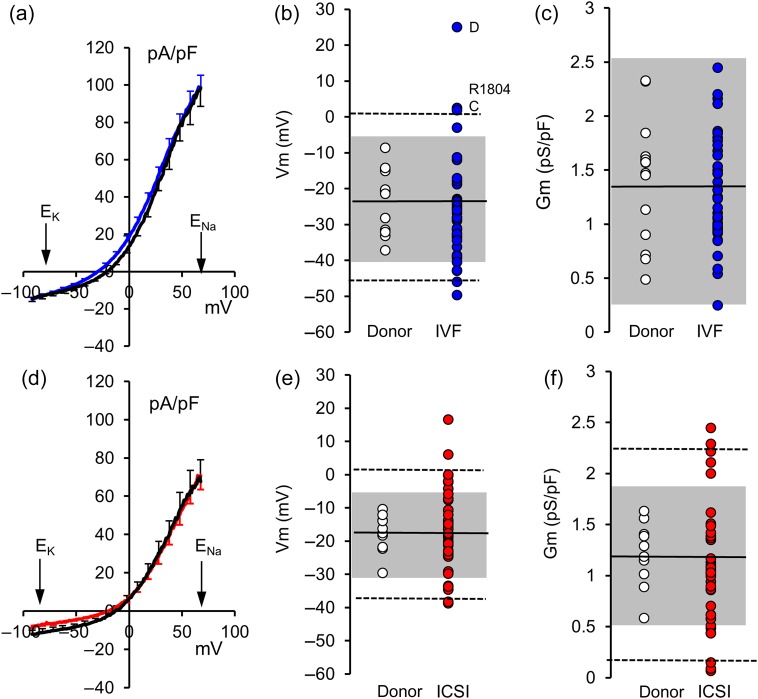
(**a**) Mean ramp-induced *I*–*V* relationship for IVF patients (blue; *n* = 40 patients) and control donor samples prepared under the same (capacitating) conditions (black; *n* = 16 donors). Error bars show ±1 SEM. Distribution of *V*_m_ (**b**) and Gm (**c**) for IVF patients (blue symbols; *n* = 40) and donor samples prepared under the same (capacitating) conditions (open symbols; *n* = 16). Black line shows donor mean, dashed lines and grey shading show 99 and 95% limits for two-tailed *T*-distribution of donor samples. Labelled points show patients illustrated in Fig. 3a and c. (**d**) Mean ramp-induced *I*–*V* relationship for ICSI patients (red; *n* = 41 patients) and donor control samples prepared under similar (non-capacitating) conditions (black; *n* = 10 donors). Error bars show ±1 SEM. (**e** and **f**) Distribution of values of *V*_m_ and Gm, respectively, for ICSI patients (red symbols; *n* = 41 patients) and donor samples prepared under similar (non-capacitating) conditions (open symbols; *n* = 10 donors). Black line shows donor mean, dashed lines and grey shading show 99 and 95% limits for two-tailed *T*-distribution of donor samples.

To compare voltage ramp-induced currents in donor and patient sperm, a mean *I*–*V* (current–voltage) curve was calculated for each individual. These were then combined to produce *I*–*V* plots for donors and for patients (IVF and ICSI). Consistent with previous findings (Mansell *et al.*, 2014), application of voltage ramps (−92 to 68 mV) to donor cells prepared under capacitating conditions (16 donors, 49 cells) elicited modest inward currents at voltages negative to −20 mV and strong outward currents at depolarized voltages. The mean response of spermatozoa from IVF patients (40 patients, 117 cells) to application of voltage ramps was similar (Fig. 1a). To quantify the characteristics of voltage ramp-induced currents, we determined zero current potentials (*V*_m_, reversal potential for the recorded currents) and conductance between 20 and 68 mV (Gm) for every cell and used these to calculate mean values in each individual. Consistent with the similarity of *I*–*V* plots, neither *V*_m_ (donor = −22.7 ± 2.0 mV; patient = −26.1 ± 2.3 mV; *P*= 0.30) nor Gm (donor = 1.41 ± 0.13 nS/pF; patient = 1.30 ± 0.08 nS/pF; *P*= 0.47) differed significantly between the donor control (*n* = 16) and IVF patient (*n* = 40) groups (Fig. 1b and c). However, among IVF patients, there was much greater variation of *V*_m_ between individuals (Fig. 1b; variance ratio = 3.12; *P*= 0.020).

Data from ICSI patients and donor controls (cells prepared under similar conditions) were analysed in the same way. For the majority of ICSI patients, very few cells were available and in most cases, records from only one cell were obtained. As with spermatozoa from IVF patients, the *I*–*V* curves for donors (10 donors, 27 cells) and ICSI patients (41 patients, 50 cells) were similar (Fig. 1d) and neither mean *V*_m_ (donor = −17.7 ± 1.8 mV, *n* = 10; patient = −16.5 ± 1.9 mV; *n* = 41; *P*= 0.67) nor mean Gm (donor = 1.21 ± 0.10 nS/pF; patient = 1.06 ± 0.09 nS/pF; *P*= 0.29) differed significantly. However, similarly to IVF patients, variability of *V*_m_ between individuals was significantly greater in the ICSI patient group than the control donor group where cells were prepared under the same conditions (variance ratio = 4.33; *P*= 0.024).

### Abnormal currents in IVF and ICSI patients

For most IVF and ICSI patients, the mean *I*–*V* curve resembled those seen in cells from controls (similarly prepared donor cells), but with more variable amplitude and reversal potential. Patients were classified as ‘abnormal’ when the mean *V*_m_ and/or Gm fell in the outer 5% of the two-tailed *T*-distribution calculated from the control (donor) data (Fig. 1). Nine out of 40 IVF patients (22%) had cells with abnormal *V*_m_ (including both depolarized and hyperpolarized cells) and 16 out of 41 ICSI patients (40%) were classified as abnormal by *V*_m_ and/or Gm (Fig. 1; Table I).

**Table I:** Summary of key parameters for spermatozoa from IVF and ICSI patients where values of *V*_m_ and/or membrane conductance (Gm; derived by regression analysis of data recorded from 20 to 68 mV) lay in the outer 5% (*), 1% (**) or 0.1% (***) of the two-tailed *T*-distribution calculated from values for donor control samples prepared under the same conditions (shown in the ‘*P*’ column).

Code	Treatment	Donors/cells	*V*_m_ (mV)	*P*-value	Gm (nS/pF)	*P*-value
Capacitated control donors		16 donors	−22.7 ± 2.0	—	1.41 ± 0.13	
R1843	IVF	1 cell	−49.67	**	2.45	
R1798	IVF	1 cell	−46.05	*	0.92	
R1856	IVF	3 cells	−42.92	*	1.83	
A	IVF	8 cells	−42.85	*	2.19	
R1870	IVF	2 cells	−40.45	*	0.69	
R1802	IVF	2 cells	−3.05	*	0.54	
C	IVF	5 cells	1.82	**	1.31	
R1804	IVF	2 cells	2.41	**	1.25	
D	IVF	13 cells	25.01	***	0.25	*
Non-capacitated control donors		10 donors	−17.7 ± 1.8	—	1.21 ± 0.10	
R1414	ICSI	1 cell	−38.8	**	0.48	*
R1848	ICSI	1 cell	−38.27	**	2.29	**
R1788	ICSI	1 cell	−33.77	*	2.11	*
R1729	ICSI	1 cell	−34.62	*	2.22	*
R1754	ICSI	1 cell	−33.54	*	1.08	
A1	ICSI	1 cell	−24.75		0.44	*
R9999	ICSI	1 cell	−24.17		2.45	**
R1873	ICSI	1 cell	−14.89		2.00	*
X	ICSI	1 cell	−12.20		0.09	**
R1640	ICSI	1 cell	−4.32	*	1.05	
R1632	ICSI	1 cell	−2.30	*	1.02	
R1777	ICSI	1 cell	−0.26	*	1.03	
R1943	ICSI	1 cell	−2.02	*	1.42	
Y	ICSI	3 cells	−0.08	*	0.15	**
K	ICSI	8 cells	5.98	**	0.08	**
R1819	ICSI	1 cell	16.54	***	0.61	

Rows for ‘capacitated control donors’ and ‘non-capacitated control donors’ show mean ± SEM for donor cells prepared under identical conditions to patient samples. Capacitated refers to samples where cells following preparation were incubated in Quinn's Advantage™ Fertilization Media and left to capacitate at 37°C/5% CO_2_ for a minimum of 3 h prior to recording. Non-capacitated refers to cells where, following preparation, they were incubated in SWM prior to recording to simulate conditions used in preparation of cells for ICSI (see the Materials and methods section).

**Figure 2:**
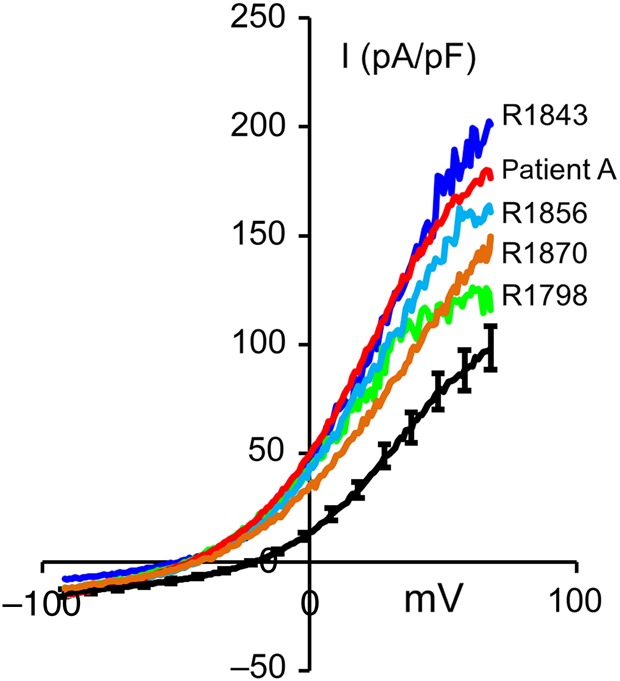
*I*–*V* traces from five IVF patients with strongly hyperpolarized *V*_m_ (<−40 mV). Black trace shows mean ± SEM *I*–*V* for donor cells (controls) prepared under identical conditions (See the Materials and Methods section).

In most cases, examination of *I*–*V* traces categorized as abnormal showed no marked abnormality consistent with loss or gross changes in characteristics of the conductances expressed. For instance, in five of the IVF patients, *V*_m_ was more negative than −40 mV, but both outward conductance and inward current at negative potentials were within the normal range (Table I). In these cells, activation of outward current apparently occurred at more negative voltages (Fig. 2). However, among the patients showing depolarization of *V*_m_, two types of abnormality were discernible.

#### Depolarization associated with low outward conductance

**Figure 3:**
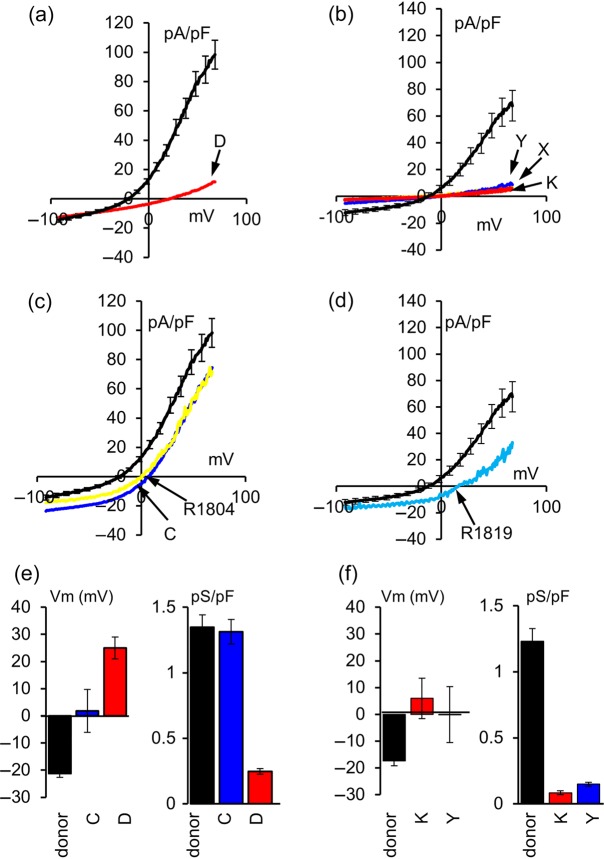
Abnormal current–voltage relationships in spermatozoa from patients. Mean *I*–*V* relationships for (**a**) one IVF patient (Patient D) and (**b**) three ICSI patients (Patients Y, X and K) in which outward conductance was negligible. Black plot shows the mean *I*–*V* (±SEM) for donor samples prepared under the same conditions. The mean *I*–*V* relationships for (**c**) two IVF patients (Patients C and R1804) and (**d**) one ICSI patients (Patient R1819) in which outward conductance was ‘normal’, but increased inward conductance caused depolarization of *V*_m_. Black lines show mean *I*–*V* (±SEM) for donor samples prepared under the same conditions (see the Materials and Methods section). (**e**) Mean ± SEM *V*_m_ (left panel) and Gm (right panel) for cells from IVF Patients C (*n* = 5 cells) and D (*n* = 13 cells) compared with mean of all control donor cells prepared under the same conditions (*n* = 49 cells). (**f**) Mean ± SEM *V*_m_ (left panel) and Gm (right panel) for cells from ICSI Patients K (*n* = 8 cells) and Y (*n* = 3 cells) compared with the mean of all control donor cells prepared under the same conditions (*n* = 27 cells).

Both IVF and ICSI populations included patients with very low outward conductance. Cells from IVF Patient D (*n* = 13 cells; Fig. 3a) had a conductance of 0.25 ± 0.02 nS/pF (*P*< 0.001 compared with mean Gm for capacitated donor cells) and positive *V*_m_ (mean=+25.0 ± 4.0 mV; Table I; Fig. 3e). In three of the abnormal ICSI patients [Y (3 cells), K (8 cells) and X (1 cell)], a similar pattern was seen. Outward conductance in cells from these patients was negligible (≈0.1 pA/pF; *P*< 0.005) compared with the mean Gm for donor cells prepared under similar conditions (Table I; Fig. 3b and f). In a separate series of experiments, we investigated the effects of high [Ca^2+^]*_i_* (50 µM) on K^+^ currents in cells from Patient K (see the Materials and Methods section). Whereas patching of cells from Patient K with pipettes containing 50 μM Ca^2+^ backfill failed to increase the outward conductance over that seen using 100 nM Ca^2+^ backfill [Gm = 0.06 ± 0.01 (*n* = 4) and 0.10 ± 0.01 (*n* = 5) with [Ca^2+^]*_i_* = 50 μM and 100 nM Ca^2+^, respectively; *P*= 0.06], recordings from donor cells, carried out in parallel, showed an increase in conductance from 0.47 ± 0.08 (*n* = 6 cells) with backfill containing 100 nM Ca^2+^ to 1.11 ± 0.03 (*n* = 4 cells) with 50 μM Ca^2+^ (*P* < 0.001).

#### Depolarization associated with enhanced inward current

Sperm from two IVF patients (C and 1804) were depolarized [+1.8 ± 7.8 mV (SEM) and +2.4 mV, respectively], but conductance was normal (Table I; Fig. 3c and e). In Patient C, where records from five cells were obtained, membrane current at *E*_K_ (−79 mV; the potential at which there is no net flow of K^+^ ions) was highly variable and significantly larger than that for donor sperm (*I*_m_ = −22.2 ± 6.5 pA/pF; *n* = 5 compared with −13.3 ± 0.9 pA/pF, *n* = 49, *P*< 0.01, Supplementary data, Fig. S1). Scatter plotting of single-cell conductance (Gm) versus *V*_m_ showed a positive relationship for this patient (*P* < 0.1; Supplementary data, Fig. S2), consistent with a major contribution of Na^+^ or non-selective permeability to the cell–cell variation in conductance. Sufficient sample from this patient was available for assessment of viscous medium penetration, a CatSper-dependent behaviour (Alasmari *et al.*, 2013b; Luo *et al.*, 2015; Williams *et al.*, 2015). Performance was comparable to donor sperm, showing a clear stimulatory effect of progesterone (data not shown). One ICSI patient (R1819; single cell) showed a similar *I*–*V* relationship with a conductance that was within the normal range but positive *V*_m_ (Fig. 3d).

### Progesterone-activated [Ca^2+^]_i_ responses in cells from abnormal IVF patients

**Figure 4:**
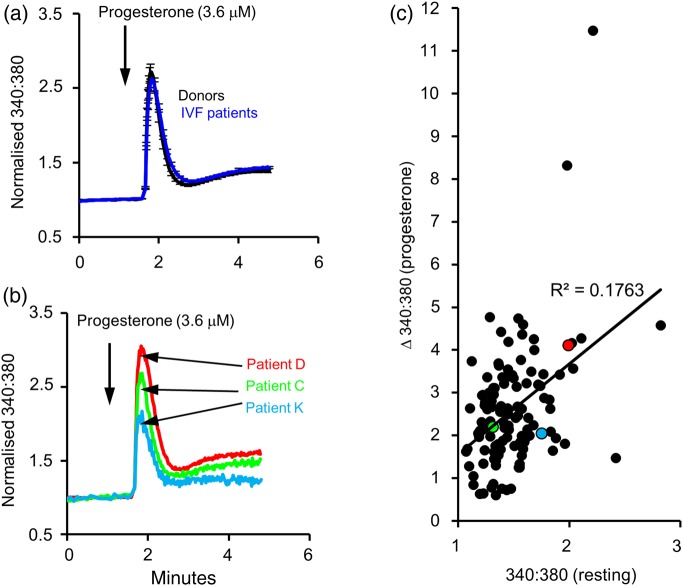
Progesterone-stimulated [Ca^2+^]*_i_* signals appear normal in patients. (**a**) Mean ± SEM [Ca^2+^]*_i_* (fura-2 340:380 ratio normalized to control period) in donor samples (black line, *n*= 45) and IVF patients (red line; *n* = 124). (**b**) Mean [Ca^2+^]*_i_* in two IVF (Patients C and D)and one ICSI patient (Patient K) in which electrophysiological investigation identified abnormalities in membrane conductance and/or *V*_m_. In both panels, the arrow shows when progesterone (3.6 μM) was added to the cells. (**c**) The relationship between the fura-2 ratio before stimulation [340:380 (resting)] and the size of the subsequent progesterone-induced increase in the ratio [Δ340:380 (progesterone)] in control donors (black symbols) and in Patients C (green symbol), D (red symbol) and K (pale blue symbol). Both the resting [Ca ^2+^]*_I_* signal and the responses of these donors fall within the normal range.

K^+^ channel function in sperm influences *V*_m_ and thus may regulate gating of CatSper. To test this, we investigated the [Ca^2+^]*_i_* response of IVF patients to progesterone (3.6 μM), which directly activates the CatSper channel. Stimulation of cell suspensions with progesterone induced a biphasic increase in [Ca^2+^]*_i_* that was virtually identical in donors (*n* = 45) and IVF patients (*n* = 124) (*P*= 0.44; Alasmari *et al.*, 2013a; Williams *et al.*, 2015). An initial [Ca^2+^]*_i_* transient, which peaked within 15 s and decayed over the following minute, was followed by a slowly developing plateau phase (Fig. 4a). In three of the abnormal patients (Patients C, D and K), there was sufficient sample available to record the [Ca^2+^]*_i_* signal induced by application of progesterone. Although resting potentials of spermatozoa from these patients were clearly abnormal, resting [Ca^2+^]*_i_* and progesterone-induced [Ca^2+^]*_i_* responses were similar to those of donor sperm and sperm from IVF patients (Fig. 4; *P*> 0.5).

### Genetic analysis of Patients D and K

Analysis of the exome sequences of Patients D and K revealed no candidate causative mutations in KCNU1 (Slo3), KCNMA1 (Slo1) or LRRC52. While no rare variants were detected, Patient K was heterozygous for SNPs rs17407838 (LRRC52, D209E, MAF A = 0.0585) and rs28608091 (KCNU1, W768R, MAF C = 0.3798). Patch clamp analysis of heterologously expressed KCNU1 constructs carrying the rs28608091 mutation indicated that it did not alter the electrophysiological properties of the channel (Supplementary data, Fig. S3). A broader analysis was performed using a candidate gene list (Supplementary data, Table SI) also failed to identify any homozygous, or compound heterozygous, candidate mutations (Supplementary data, Table SII).

### Biophysical abnormality and fertilizing potential of spermatozoa IVF

**Figure 5:**
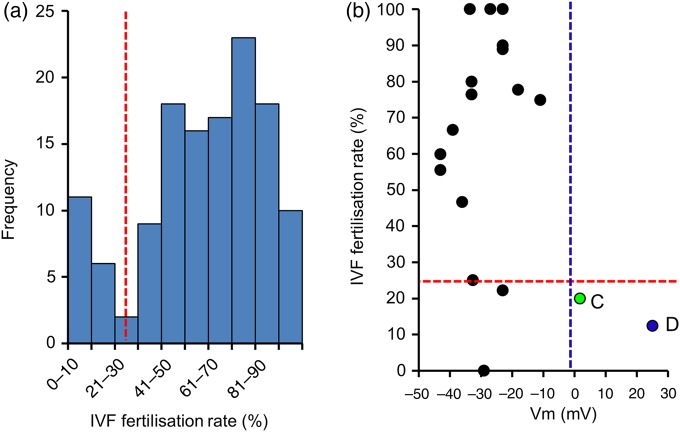
Highly depolarized membrane potential is associated with poor fertilization rate in IVF. (**a**) The distribution of fertilization rates (% fertilized) for 126 IVF patients. Two separate groups can be identified (separated by dashed red line). Most patients achieved ≥25% fertilization but a ‘poor’ subgroup, with fertilization rate <25% is also present. (**b**) The relationship between mean membrane potential and fertilization rate for 19 patients for whom multiple recordings and fertilization rate data were obtained. (Patients C and D are labelled.) Dashed lines delineate categories used for *χ*^2^ contingency analysis: red line shows poor and normal fertilization groups (<25 and ≥25%); blue line shows ‘normal’ *V*_m_ and highly depolarized (<0 and ≥0 mV). *P* = 0.012.

A key question to address is the functional consequences of these abnormalities. Since IVF patient cells used for electrophysiology were aliquots from the ejaculate used on the day of IVF treatment, we were able to directly compare biophysical characteristics with fertilization success in the same ejaculate (8–20 oocytes per patient). Compiled fertilization data from a large sample of IVF patients (*n* = 126) at the clinic showed a bimodal distribution (Fig. 5a) with a major peak at 70–80% (normal group) but also a discrete group of patients with abnormally poor fertilization (0–25%). Using the IVF data from 19 patients where we had current records from ≥3 cells (providing an acceptable estimate of population characteristics), we investigated the relationship between mean *V*_m_ and fertilization rate. Complete depolarization of *V*_m_ (≥0 mV; Patients C and D) was significantly associated with the group showing poor fertilization rates (≤25%) (Fig. 5b; *P* = 0.016; *χ*^2^ contingency).

## Discussion

In the present study, we have used the whole-cell recording technique to characterize the biophysical properties of sperm from 81 subfertile patients undergoing IVF/ICSI. This is by far the most extensive such study ever undertaken. In both IVF and ICSI patients, the mean *I*–*V* relationship was similar to that for donor controls. However, the variability of biophysical characteristics (*V*_m_ and Gm) between individuals was much greater among patients than controls (donors) and in a significant minority of patients (20–40%), one or more biophysical characteristics were abnormal. In patients recommended for ICSI treatment, multiple abnormalities of spermatogenesis are common, manifested as reduced sperm count, morphology and/or motility (Sakkas *et al.*, 2015). The detection of ion channel malfunction in these men is therefore perhaps not surprising, although the high incidence of biophysical abnormalities is nevertheless striking. However, patients selected for IVF had effectively ‘normal’ semen parameters (WHO, 2010) and the occurrence of biophysical abnormalities in up to 20% of these men supports the hypothesis that ion channel malfunction may contribute significantly to idiopathic male infertility.

### Characteristics of currents in patient spermatozoa

Although the occurrence of greater biophysical variability in spermatozoa of patients must reflect, in part, the presence of some patients with clear abnormalities of current characteristics (see below), there also appeared to be an ‘exaggeration’ of the innate variability in conductance and reversal potential that was present in the control (donor) group. Outward currents recorded under the quasi-physiological conditions used here are carried primarily by flux through a conductance that is poorly K^+^ selective with *G*_K_:*G*_Na_ ≈7 (Mansell *et al.*, 2014). This channel shows <25% activation when *V*_m_ < 0 mV and although reversal potentials for cells prepared under both capacitating and non-capacitating conditions are closer to *E*_K_ (−79 mV) than *E*_Na_ (+69 mV), it is clear that the flux of ions other than K^+^ contributes to resting *V*_m_. Since incubating cells under IVF capacitating conditions results in a change in current characteristics, it is possible that uncoupling and/or poor regulation of the changes in channel function that occur during capacitation may result in the observed increase in biophysical variability in the patient population.

### Patients with abnormal currents

Examination of *I*–*V* records revealed at least two types of significant current abnormality. In four patients (ICSI patients X, Y, K and IVF Patient D), outward conductance was very low or negligible. Cells in these samples were depolarized and in Patients D and K (13 cells and 8 cells, respectively), the mean *V*_m_ was markedly positive (+25 and +6 mV, respectively), resembling *V*_m_ after block of K^+^ conductance with quinidine or bupivacaine (Mansell *et al.*, 2014). In spermatozoa from Patient K, we investigated the effect of elevating intracellular Ca^2+^ to 50 μM, which enhances K^+^ currents (Mannowetz *et al.*, 2013; Brenker *et al.*, 2014). This manipulation did not ‘rescue’ resting *V*_m_ or outward conductance. It is noteworthy that although most of these patients are in the ICSI group, Patients X and Y had basic semen characteristics (≥15 million cells/ml and ≥25% motility) that would be classed as close to normal by WHO standards (WHO, 2010). We conclude that in these four patients, expression and/or function of K^+^ conductance is severely impaired.

A second, characteristic abnormality (seen in two IVF patients and one ICSI patient) was the occurrence of an increased inward current such that *V*_m_ was depolarized, despite the presence of outward conductance within the normal range. In IVF Patient C, where recordings from five cells were obtained, inward current at *E*_K_ (−79 mV; where there is no net K^+^ flux) was highly variable and significantly greater than in controls. It appears that an inward leak conductance (probably Na^+^) contributed excessively to determination of *V*_m_ in these patients. Evidence for expression of epithelial sodium channels (ENaC) and voltage-gated sodium channels (Na_V_) in human spermatozoa has been reported (Kong *et al.*, 2009; Pinto *et al.*, 2009; Escoffier *et al.*, 2012; Cejudo-Roman *et al.*, 2013), but to date, no such currents have been shown in electrophysiological examination of ejaculated cells. If these channels function primarily in spermatogenic cells, incomplete and/or impaired spermatogenesis (or epididymal maturation) might result in over-expression of these conductances in ejaculated cells of these patients.

### Biophysical abnormalities and failure of sperm at IVF

A key question is, what are the functional consequences of these abnormalities? Fertilization rates of IVF patients were distributed bi-modally with a ‘normal’ group which typically achieved 70–80% oocyte fertilization and a subset where fertilization was 0–25%. Of the 19 IVF patients where currents were recorded from ≥3 cells (allowing assessment of population characteristics), only Patients C and D fell in the low fertility group (20% and 12.5% IVF fertilization rate, respectively). Spermatozoa from these patients differed fundamentally from control donor spermatozoa in their biophysical characteristics. Both were strongly depolarized, with the mean *V*_m_ > 0 mV, but whereas Patient D was effectively devoid of outward current, in Patient C, the positive *V*_m_ reflected the presence of a large inward (probably Na^+^) leak (see above). *χ*^2^ analysis showed that this association of positive membrane potential with the low IVF fertilization rate was non-random (*P* < 0.02). These data suggest that a strongly depolarized membrane potential (≥0 mV), irrespective of the underlying lesion(s), has a significant adverse effect on fertilizing ability.

A surprising finding was that although major, functionally significant (poor fertilizing potential) abnormalities of outward conductance and membrane potential regulation were detected in sperm from some patients, these sperm appeared normal in other assessments employed in this study. For instance, it has been hypothesized that sperm membrane potential and its regulation might be expected to influence CatSper function (Brenker *et al.*, 2014), yet the progesterone-induced [Ca^2+^]*_i_* signal in patients (C, D and K) resembled those of controls (donors) both in ‘normal’ patients (as described previously; Alasmari *et al.*, 2013a) and in patients with significantly abnormal conductance and/or depolarized *V*_m_. This implies that there is only a weak interaction between *V*_m_ and CatSper function. Similarly, sperm from Patient C penetrated viscous media to the same degree as controls (donor spermatozoa) both in an unstimulated state and stimulated with the CatSper agonist progesterone. Additionally, despite the apparent loss of K^+^ conductance and positive *V*_m_ recorded from cells of Patient D, kinematic parameters of sperm motility from this patient (assessed by CASA; data not shown) appeared normal, both in terms of mean value and distribution of single-cell values. Why might these spermatozoa perform normally in these tests even with highly abnormal *V*_m_? One possibility is that the assays employed here are inadequate to detect the subtle interactions between membrane potential, CatSper activity and K^+^ channel function (Brenker *et al.*, 2014). For instance, CatSper conductance in the absence of progesterone is low even at very depolarized potentials (Lishko *et al.*, 2011), but the saturating concentrations of progesterone used here may produce robust [Ca^2+^]*_i_* signals in human sperm irrespective of *V*_m_. Consequently, future studies on sperm with impaired regulation of *V*_m_ should address more subtle aspects of sperm function such as dose-dependence of the action of progesterone, the Ca^2+^-sensitivity of the K^+^ channel in human spermatozoa and its potential interplay with CatSper (Brenker *et al.*, 2014). It will be interesting specifically to investigate long-term patterns of [Ca^2+^]*_i_* and motility following CatSper activation in K^+^-channel null/impaired cells.

We can only speculate as to the nature of the abnormalities(s) that result in altered currents reported here. The characteristics of Patient C and D with reduced fertilization rate are similar to the abnormalities shown in *LRRC52* KO mice with significantly reduced but not absent fertilization rates (Zeng *et al.*, 2015). No genetic abnormalities in *SLO1*, *SLO3* or *LRCC52* genes were shown in Patient D (Patient C did not give permission for genetic analysis). It is possible that in the absence of any overt genetic abnormality, a defect(s) in testicular and/or epididymal sperm maturation and/or potential processing in the mature cell during capacitation may be present. However, although impaired localization and/or assembly of the SLO1/SLO3/LRCC52 complex is a likely explanation for loss of function, detailed proteomic studies combined with high quality co-localization and imaging will be required to address this.

In this study, we assessed the presence and prevalence of abnormalities in membrane conductance and maintenance of *V*_m_ in spermatozoa from 81 patients attending for IVF (normal in their semen characteristics, WHO, 2010) and ICSI treatment. In most cases, these spermatozoa had a *V*_m_ and current profile similar to that seen in healthy volunteer donors, although there was greater variation in both outward conductance and *V*_m_. This is consistent with a shift in channel regulation due to changes in spermatogenesis and/or epididymal maturation. In a surprisingly large number of patients, *V*_m_ and/or conductance were significantly abnormal, including a subset (5–10% of both IVF and ICSI patients) where currents were grossly abnormal. Where these abnormalities resulted in a strongly depolarized (+ve) *V*_m_, the cells were functionally compromised. Overall, these data suggest that (i) impaired K^+^ conductance and/or regulation of *V*_m_ in spermatozoa is a relatively common feature both in ICSI patients and in men classified as suitable for IVF and (ii) such lesions, particularly when they cause loss or reversal of membrane polarization, may contribute to a significant loss of fertilizing potential. This knowledge could be helpful for the development of novel diagnostic tools to assess semen quality using membrane permeable voltage-sensitive dyes.

## Supplementary data

Supplementary data are available at http://humrep.oxfordjournals.org/.

Supplementary Data
