# Supplementary material for: Depolarization of sperm membrane potential is a common feature of men with subfertility and is associated with low fertilization rate at IVF
Source: Hum Reprod. 2016 Apr 6;31(6):1147–57. doi: 10.1093/humrep/dew056 (PMC4871192; doi:10.1093/humrep/dew056)
Supplement: Supplementary Data [file supp_31_6_1147__index.html]

Depolarization of sperm membrane potential is a common feature of men with subfertility and is associated with low fertilization rate at IVF — Supplementary Data 

# Depolarization of sperm membrane potential is a common feature of men with subfertility and is associated with low fertilization rate at IVF

## Supplementary Data

Supplementary Data

- Supplementary Figure 1 - pdf file
- Supplementary Figure 2 - pdf file
- Supplementary Figure 3 - pdf file
- Supplementary Table 1 - pdf file
- Supplementary Table 2 - pdf file
