# Supplementary material for: Depolarization of sperm membrane potential is a common feature of men with subfertility and is associated with low fertilization rate at IVF
Source: Hum Reprod. 2016 Apr 6;31(6):1147–57. doi: 10.1093/humrep/dew056 (PMC4871192; doi:10.1093/humrep/dew056)
Supplement: Supplementary Data [file supp_dew056_dew056supp_fig1.pdf]

Human Reproduction, 2016
doi:10.1093/humrep/dew056

human reproduction

SUPPLEMENTARY DATA

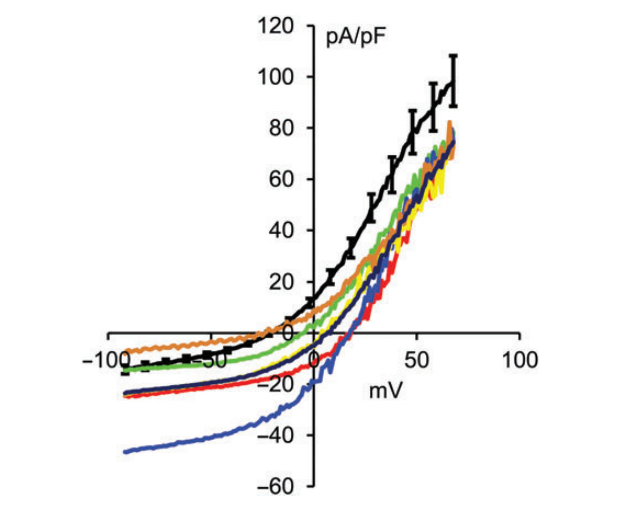

Supplementary Figure S1 Cells from Patient C vary in their currents induced by ramp depolarization. Mean donor sperm data are shown in black for reference.
