# Supplementary material for: Depolarization of sperm membrane potential is a common feature of men with subfertility and is associated with low fertilization rate at IVF
Source: Hum Reprod. 2016 Apr 6;31(6):1147–57. doi: 10.1093/humrep/dew056 (PMC4871192; doi:10.1093/humrep/dew056)
Supplement: Supplementary Data [file supp_dew056_dew056supp_fig2.pdf]

Human Reproduction, 2016
doi:10.1093/humrep/dew056

human reproduction

SUPPLEMENTARY DATA

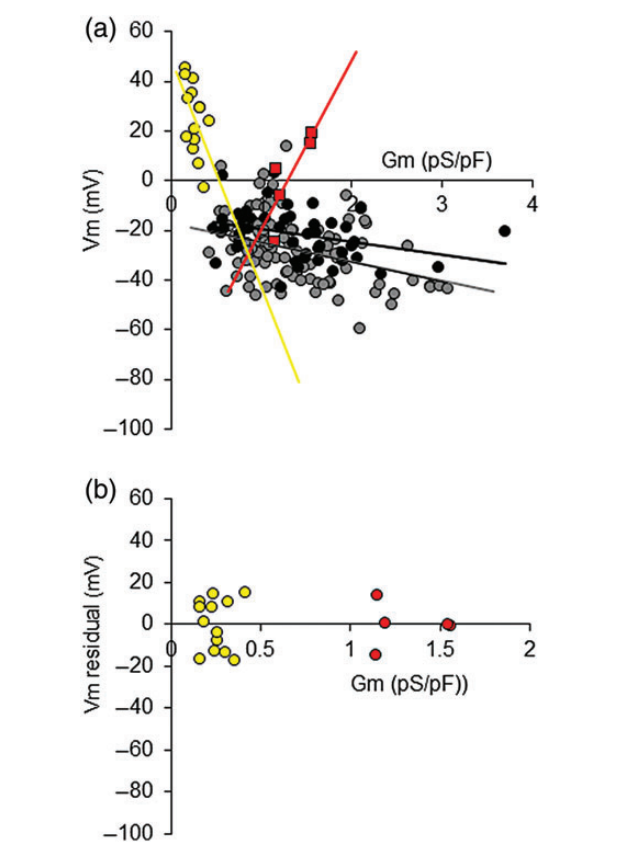

Supplementary Figure S2 (**a**) Scatter plot of membrane conductance (Gm) versus resting membrane potential (V_m) for individual cells from capacitated donors (black symbols), Patient C (red symbols), Patient D (yellow symbols) and all other IVF patients (grey symbols). Regression lines are presented in matching colours ($P < 0.1$, Patient C). (**b**) Residual plots for regressions fitted to data from patients C (red) and D (yellow). Points are randomly scattered consistent with a linear fit.
