# Supplementary material for: Depolarization of sperm membrane potential is a common feature of men with subfertility and is associated with low fertilization rate at IVF
Source: Hum Reprod. 2016 Apr 6;31(6):1147–57. doi: 10.1093/humrep/dew056 (PMC4871192; doi:10.1093/humrep/dew056)
Supplement: Supplementary Data [file supp_dew056_dew056supp_fig3.pdf]

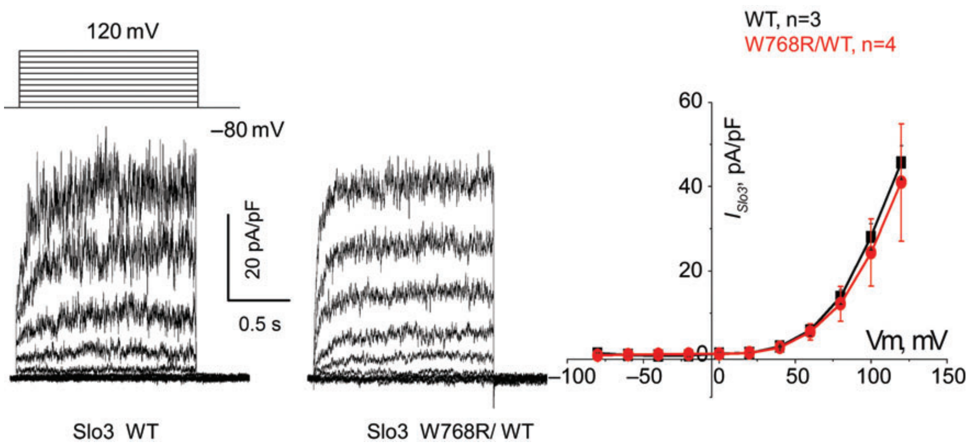

Supplementary Figure S3 Mutation of Slo3 does not affect the K^+ current from heterologously (HET) expressed Slo3 in HEK293 cells. Representative Slo3 currents in response to indicated voltage steps, recorded from HEK293 cells transfected with a WT Slo3 (left panel) or with a mixture of WT and Mutant (W768R) Slo3 (middle panel). All transfection was done in combination with LRRC52. (Right panel) Voltage to current relationship of K^+ current generated. Error bars indicate SEM. Solutions used in this experiment were all symmetrical K^+ with a pH of 7.4 (pipette and bath) and a free internal calcium concentration of 100 nM and a 100 μM external free calcium concentration.
