# Supplementary material for: Depolarization of sperm membrane potential is a common feature of men with subfertility and is associated with low fertilization rate at IVF
Source: Hum Reprod. 2016 Apr 6;31(6):1147–57. doi: 10.1093/humrep/dew056 (PMC4871192; doi:10.1093/humrep/dew056)
Supplement: Supplementary Data [file supp_dew056_dew056supp_table1.pdf]

Supplementary Table SI **Comparison of candidate loci list to the human sperm proteome.**

Gene	Sperm proteome
ATP1B1	Yes
CACNA1C	No
CACNA1D	No
CACNA1S	No
CATSPER1	Yes
CATSPER2	Yes
CATSPER3	No
CATSPER4	Yes
CATSPERB	Yes
CATSPERD	Yes
CATSPERG	Yes
CAV1	No
CNBD2	No
CTNNA1	Yes
CTNNA2	No
CTNNA3	No
CTNNAL1	No
DMD	Yes
HSP90AA1	Yes
HSP90AB1	Yes
HSP90B1	Yes
KCNMA1	No
KCNMB1	No
KCNMB2	No
KCNMB3	No
KCNMB4	No
KCNU1	Yes
LRRC52	Yes
PDZRN3	No
PDZRN4	No
PRKACA	Yes
PRKACB	No
PRKAR1A	Yes
PRKAR1B	Yes
PRKAR2A	Yes
PRKAR2B	Yes
PRKG2	No
PRKX	Yes
TMCO1	Yes
VCL	Yes
YWHAB	Yes
YWHAE	Yes
YWHAG	Yes
YWHAH	Yes
YWHAZ	Yes

A candidate data list was prepared using the String 10 database (http://string-db.org/). This list was built around known Potassium handling systems in sperm, KCNU1, KCNMA1 and LRRC52. While the full list was used to query the variants derived from the exome data set, not all candidates have been reported in the human sperm proteome (Baker MA, Naumovski N, Hetherington L, Weinberg A, Velkov T, Aitken RJ. (2013) Head and flagella subcompartmental proteomic analysis of human spermatozoa. *Proteomics*. **13**:61–74).
