# Supplementary material for: Depolarization of sperm membrane potential is a common feature of men with subfertility and is associated with low fertilization rate at IVF
Source: Hum Reprod. 2016 Apr 6;31(6):1147–57. doi: 10.1093/humrep/dew056 (PMC4871192; doi:10.1093/humrep/dew056)
Supplement: Supplementary Data [file supp_dew056_dew056supp_table2.pdf]

Supplementary Table SII **Genetic analysis of Patients D and K.**

Patient	Gene	SNP db ID	Ref	Allele	GMAF	Zygosity
D	CATSPER3	rs114447625	G	A	A:0.0086	Heterozygote
D	PRKAR1B	rs61732492	T	C	C:0.0186	Heterozygote
D	DMD	rs1800279	T	C	C:0.0148	Hemizygote
K	CATSPER4	rs12138368	G	T	T:0.0234	Heterozygote
K	CATSPER2	rs2614835	T	C	C:0.0004	Heterozygote

SNP and Indel identification was performed using a standard GATK workflow following bowtie alignment (McKenna *et al.*, 2010; DePristo *et al.*, 2011; Van der Auwera *et al.*, 2013). The GATK output was filtered against the candidate gene list given in Table I. SNPs/Indels outside coding regions (REFSEQ) and those encoding synonymous changes or with a minor allele frequency greater than 0.05 (as per 1000 Genomes, http://www.1000genomes.org/) were excluded.
